# Two roads diverged in multiple sclerosis: When is switching therapy effective?

**DOI:** 10.1016/j.neurot.2025.e00734

**Published:** 2025-09-05

**Authors:** Anthony T. Reder

**Affiliations:** Department of Neurology MC-2030, University of Chicago Biological Sciences Division, Chicago, IL, USA

There are 24 FDA-approved therapies for multiple sclerosis (MS) with 12 different mechanisms of action in this complex disease. All therapies are most effective in early relapsing/remitting MS (RRMS). However, none is perfect, as some patients on therapy have exacerbations, new MRI lesions, disease progression, or side effects.

In this issue of Neurotherapeutics, Chisari et al. studied the benefit and safety of switching to ofatumumab after disease activity or intolerance to other therapies [1]. After a year of monthly subcutaneous injections of this anti-CD20 monoclonal antibody, patients were free of new MRI lesions (85 ​%), relapses (93 ​%), and progression (94 ​%), nearly identical to therapy-naïve patients who started this therapy. Surprisingly, the subgroup that switched from “moderate efficacy” dimethyl fumarate, teriflunomide, interferon-beta, and glatiramer had fewer new MRI lesions at 12 months than those who switched from several “high efficacy” therapies. The significantly worse profile in the high efficacy subgroup was driven by switches from cladribine, fingolimod, and alemtuzumab; switches from natalizumab did well. Note, the term “high-efficacy” is based on reduction in relapses; effects on MRI and on progression independent of relapses are variable or differ minimally between therapies.

This is a non-randomized “real-world” study, potentially confounded by reasons for switching, by different durations of therapy, and by prescribing bias, *i.e.*, high efficacy drugs are used for perceived severe MS. For instance, 90 ​% of natalizumab-treated patients were clinically stable but had become JC virus antibody-positive. In contrast, the other high efficacy drugs all had breakthrough MS activity. However, patients with breakthrough activity on moderate efficacy drugs did well after switching. There may be a biological basis for these results.

MS drugs can have long-term consequences on immune function. Prior chemotherapy increases the conversion rate to JCV ​+ ​status four-fold during natalizumab treatment, and greater numbers of prior MS therapies increase the risk of cancer [2]. Cladribine, anti-CD20 antibodies, alemtuzumab, and S1PR modulators such as fingolimod, modify immunity for years after treatment; five years of interferon-β therapy prolongs longevity by seven years [3]. At least four therapies reverse subnormal regulatory CD8 T cells (Interferon, glatiramer acetate, anti-CD20, and S1PR modulators, in Ref. [4]) or Th2 cells (glatiramer [5]). Long-term effects are unexplored.

The immune environment itself is abnormal before onset of MS. Later-in-life polio infection causes paralysis; late EBV infection is linked to MS onset years afterward [6]. Compared to healthy controls, mononuclear cells from untreated RRMS patients have abnormal expression of 8000 genes, significant dysregulation of alternative RNA splicing, and severely imbalanced levels of serum cytokines [[7], [8], [9]]. In this light, current MS therapies are likely to alter the global gene and protein disruption seen in early inflammatory MS. Because they have different mechanisms of action, they will have different long-term consequences. The immune environment also changes over time during ongoing MS. In secondary progressive MS, abnormal gene expression diminishes to 3600 genes, with new abnormalities in repair pathways [10]. This may explain why current therapies have only partial effect on progression.

The immune environment in MS changes with disease duration, patient age, and the legacy of prior therapy. All are important in clinical sequencing of therapies, at different ages, and in different forms of MS. Chisari et al. illustrate that the choice between therapies at a fork in the therapeutic road must be evaluated with an eye on patient characteristics as well as on prior therapies.

## Author contribution

ATR wrote the editorial, without using AI.

## Declaration of competing interest

The authors declare the following financial interests/personal relationships which may be considered as potential competing interests:Anthony T Reder reports was provided by University of Chicago Medicine. Anthony T Reder reports a relationship with Bristol Myers Squibb Co that includes: funding grants. Anthony T Reder reports a relationship with Genentech USA Inc South San Francisco that includes: consulting or advisory and funding grants. Anthony T Reder reports a relationship with Sanofi that includes: consulting or advisory. Given his role as reviewer, ATR was involved in the peer review of this article. If there are other authors, they declare that they have no known competing financial interests or personal relationships that could have appeared to influence the work reported in this paper.
